# Persistent Neutrophilic Inflammation is Associated with Delayed Toxicity of Phenylarsine Oxide in Lungs

**DOI:** 10.21203/rs.3.rs-5100050/v1

**Published:** 2025-01-13

**Authors:** Nilda C. Sanchez, Gopikrishnan Mani, Carlin Jones, Jaroslaw W. Zmijewsli, Ranu Surolia

**Affiliations:** University of Alabama at Birmingham; University of Alabama at Birmingham; University of Alabama at Birmingham; University of Alabama at Birmingham; University of Alabama at Birmingham

**Keywords:** Phenyl Arsine Oxide, Arsenicals, Neutrophil extracellular traps, IL-33, persistent inflammation, Airway remodeling

## Abstract

Phenyl arsine oxide (PAO) is a vesicant, similar to Lewisite, a potential chemical warfare agent and an environmental contaminant. PAO-induced skin burns can trigger acute organ injury, including lungs. We have recently demonstrated that PAO burns can also has a delayed toxicity, although the specific mechanism/s remain to be determined. A single cutaneous exposure to PAO resulted in inflammatory acute lung injury at 6 and 24 hours. While acute injury subsiding by 1 week, we observed a significant airway remodeling at 10 weeks post-PAO exposure. The mechanism of prolonged PAO toxicity was associated with the influx of neutrophils that produced harmful neutrophil extracellular traps (NETs). We demonstrated that the crosstalk between NET deployments and expression of IL-33, a pro-remodeling mediator was associated with the development of peribronchial fibrosis. In summary, these results suggest that a single cutaneous exposure to PAO causes the acute inflammatory phase followed by NETs/IL-33 feed forward signaling implicated for the persistent neutrophil influx and NETs formation resulting in airway remodeling.

## Introduction

Lewisite, a chemical warfare agent containing organoarsenic, was developed and deployed during the World Wars I and II [1]. After the wars, more than 40 countries disposed of their Lewisite stockpiles in the sea [2]. According to a recent report, levels of lewisite and its degradation product; phenyl arsine oxide (PAO) are rising in the Baltic Sea at its munition sites [3], which is a significant concern for environmental scientists [4, 5]. Also, the unmarked buried sites, accidental exposures and intentional unethical use of this chemical agent is among the risk factors for the exposure to lewisite, and its degradation product PAO [6].

PAO, a trivalent organic compound with vesicant properties similar to Lewisite, is a potential chemical warfare agent and an environmental contaminant. For the first time, our group published a mechanistic study in which we demonstrated that single cutaneous exposure to PAO led to acute lung injury [7]. In our follow up studies, we also found that a single skin exposure to a sub-lethal dose of PAO was sufficient to demonstrate delayed lung injury effects in 10 and 20 weeks after exposure and led to the pathogenesis of constrictive bronchiolitis (CB) in mice [7]. CB in the mice lungs was similar to the CB presented in the survivors of sulfur mustard, a similar organoarsenic vesicant CWA, attacks during the Iran-Iraq War [8]. However, the mechanism of the delayed pulmonary toxicity of cutaneous exposure to PAO is yet unknown. In this study, we aim to investigate the mechanisms of delayed pulmonary toxicity posed by cutaneous PAO exposure.

In our PAO induced acute lung injury (PAO-ALI) model, we demonstrated that the acute yet the systemic injury to the lungs was triggered by neutrophil inflammation and netosis. Neutrophils have short life span, and specifically, netosis is the suicidal death of neutrophils, where the activated neutrophils spill out the chromatin DNA in the extracellular space [9]. These extracellular chromatin DNA strands are called neutrophil extracellular traps (NETs), which are rich in neutrophil elastase and MPO content and, in excess, cause tissue damage. CB is a chronic inflammatory disease of the airways, where repeated injury of airway epithelial cells leads to fibrotic constriction and/or pruning of small airways [10]. The molecular mechanism of this disease is not well studied. Interestingly, deployment-related constrictive bronchiolitis (DRCB), linked to toxic exposures such as burn pit fumes, is associated with sustained NET formation in the lungs of affected individuals [11]. Based on these important observations, we performed the time-dependent single cutaneous PAO exposure in mice to understand the inflammation and its role in delayed PAO toxicity.

## Results

### PAO exposure on the skin triggers ALI, and inflammation mitigates after a week.

**Figure 1:**
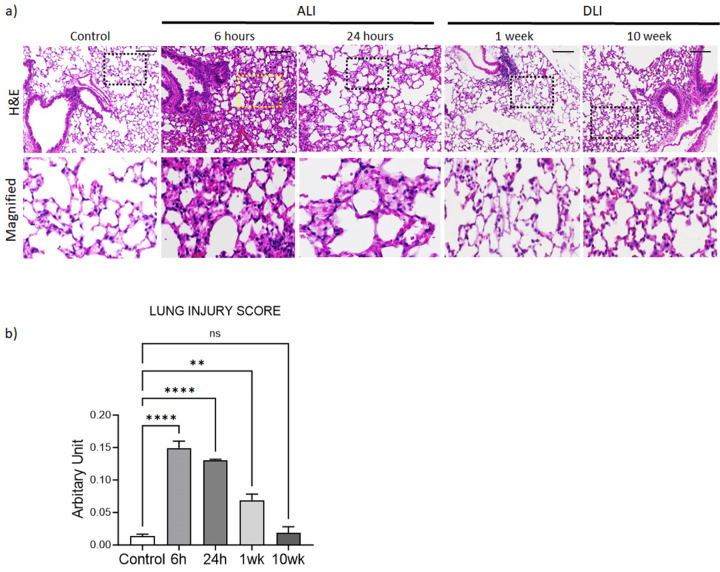
Cutaneous exposure to PAO induces acute and delayed lung injury in mice. Mice (*n*=3–5 per group) were exposed to PAO (0 or 150 mM) for 6, 24 hours, or 1 and 10-weeks. (**a**) Upper panel indicates a representative H&E staining of the lung sections from indicated groups of mice, while lower panel shows enlarged regions of lungs indicated by dashed boxes. Scale bar 100 mm. (**b**) Lung Injury Score is shown. Data presented as mean ± SEM (*n* = 3–5 mice per group), ****p<0.0001, ANOVA, control *vs*. PAO exposure. *ALI*- acute lung injury; *DLI*-delayed lung injury.

The histopathological analysis of lung tissues involved using hematoxylin and eosin (H&E) staining. The groups exposed to PAO at 6 hours and 24 hours post-exposure exhibited a significant increase in lung tissue cellularity. The lungs at these time points showed a higher presence of proteinaceous debris in the airspaces, indicating acute lung injury (Fig. 1a, ALI insets). However, at 1 week mice demonstrated reduced inflammation (Fig. 1a, DLI). The assessment of lung injury scores according to the American Thoracic Society guidelines [12] indicated significant lung injury in the 6-hour, 24-hour (p < 0.0001, control *vs*. PAO), and 1-week exposure samples (p < 0.001, control *vs*. PAO) (Fig. 1b). However, the 10-week groups showed insignificant differences in the lung injury scores, compared to the control (Fig. 1b).

### PAO exposure displays neutrophilic inflammation in the lungs.

**Figure 2:**
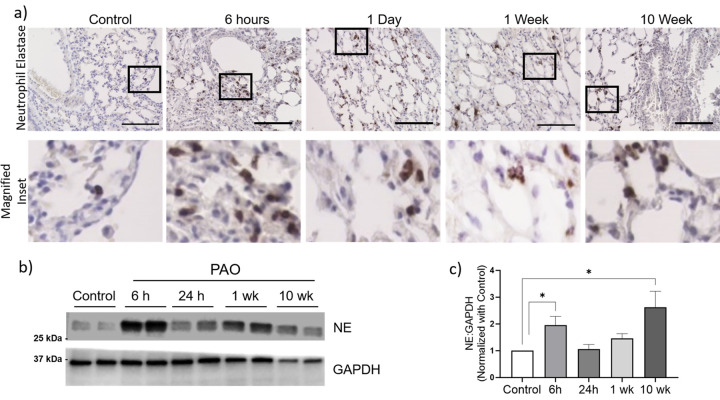
PAO-induced neutrophil-dependent inflammation in the lungs. (a) Representative images of lung sections show staining of neutrophil elastase (NE) in control and PAO exposure groups (n=3). Scale bar 100 μm. (b and c) Immunoblot analysis of the whole lung homogenates for NE and GAPDH. Data normalized to the loading control GAPDH. Data are presented as mean ± SEM (n = 3) *P <0.05, ANOVA, as compared PAO to the control group of mice.

Acute lung injury is characterized by intense neutrophilic inflammation. In our study, we conducted immunohistochemistry of lung sections to detect the presence of neutrophils. Our analysis revealed a significant increase in neutrophil influx in the lungs of mice exposed to PAO at 6 hours and 24 hours post-exposure compared to the control group (Fig. 2a). Additionally, the mice at 1 week and 10 weeks post-exposure; also displayed elevated numbers of neutrophils in the lung interstitium. We observed magnified areas of anti-NE stained neutrophils but did not find an increased number of neutrophils in the alveolar spaces upon close examination (Fig. 2a, inset). Furthermore, whole lung lysate analysis for the expression of neutrophil elastase demonstrated increased expression in the PAO-exposed mice compared to the control group (Fig. 2b). Densitometry analysis demonstrated that PAO exposed 6-hours and 10-weeks mice groups exhibited increased neutrophil elastase levels (Fig. 2c), while the PAO exposed 24-hour and 1-week groups showed reduced expression levels of neutrophil elastase (Fig. 2b–c).

### A single cutaneous exposure to PAO instigates persistent presence of NETs in the lungs.

**Figure 3:**
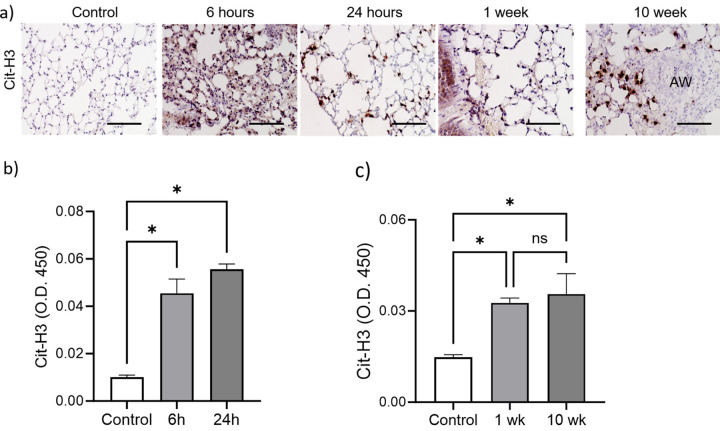
Persistent NETosis in lungs of PAO-exposed mice. (**a**) Representative images shows NETosis primed neutrophils and diploid NETs using immunostaining for citrullinated histones 3 (Cit-H3) in lungs of control *vs*. PAO-treated groups of mice (*n*=3/group). Scale bar 100 mm. (**b-c**) Western blot analysis of Cit-H3 in lung homogenates from control and PAO-exposed mice (*n*=3–5 per group). **d**) Western blot analysis of IL-33 in lung homogenates from indicated groups of mice (*n*=3–5). All data are presented as mean ± SEM, (ns) non-significant, *p < 0.05, **p < 0.01, and ***p < 0.001, ANOVA, as compared to control.

We analyzed the lungs of mice exposed to a single dose of PAO cutaneously for the increased neutrophilic inflammation in terms of netosis. Netosis is an inflammatory phenomenon where the activated neutrophils spill DNA traps in the extracellular space. Citrullinated histone 3 (Cit-H3) is attached to this extracellular DNA and is utilized as a netosis marker. The immunohistochemistry of the lung section for Cit-H3 demonstrated the presence of NETs in all the PAO-exposed exposure groups (Fig. 3a). The control group had an absence of NETs.

The 6-hour and 24-hour PAO-exposed mice groups demonstrated an approximately 3-fold increase in Cit-H3, as compared to controls (Fig. 3b). The 1-week and 10-week PAO-exposed mice groups shown significantly increased levels of Cit-H3 (*p < 0.05, control vs. PAO-exposed groups) (Fig. 3c).

IL-33 is a pro-netotic ‘alarmin’ cytokine [13]. 6-hour, 24-hour, 1-week and 10-week PAO-exposed mice groups demonstrated significantly elevated levels of IL-33 compared to controls in their whole lung lysates (Fig. 3d).

## Single cutaneous exposure to PAO induced airway remodeling

**Figure 4:**
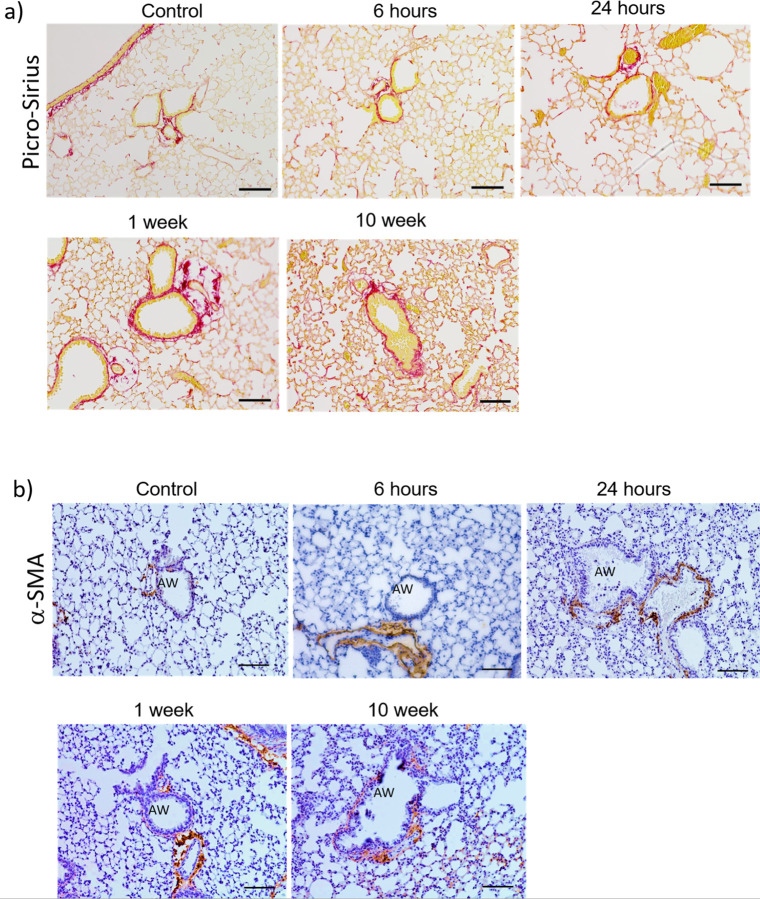
A single cutaneous exposure to PAO causes delayed airway remodeling after one or 10 weeks. (**a**) Representative images show lung sections stained with Picro Sirius, while (**b**) depicted the levels of a-SMA positive cells within the airways of PAO-treated mice. Data obtained from *n*=3 mice per group. Scale bar 100 mm. AW = airway.

The histological analysis demonstrated the presence of restrictive airways at 1-week and 10-weeks post-PAO exposure. There were changes observed in the collagen fibers, and α-SMA deposition around airways after 6 and 24 hours of cutaneous PAO exposure (Fig. 4a,b). The 1-week PAO-exposed mice group showed increased collagen around the airways; however, no changes were observed in the thickness of α-SMA around the airway (denoted as AW in the image). The 10-week PAO-exposed mice group showed increased deposition of collagen fibers and the increased thickness of α-SMA around the airways (Fig. 4a,b).

### PAO-induced IL-33; but not the pro-fibrotic SMAD3 expression in primary human airway epithelial cells

**Figure 5:**
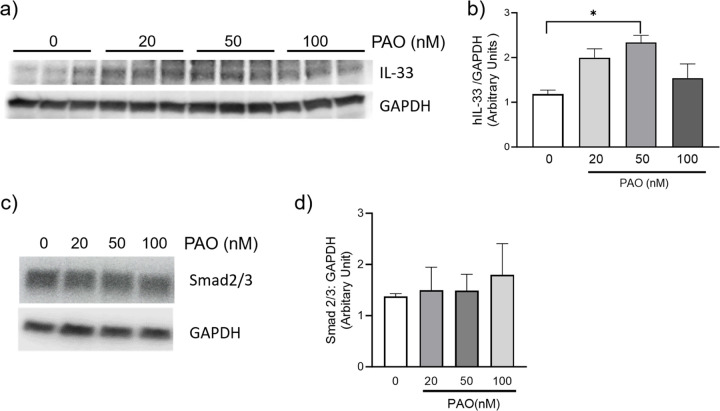
PAO-induced production of IL-33 in BEAS-2B cells. (**a-b**) Immunoblot analysis of hIL-33/GAPDH in lysates from BEAS-2B cells that were treated with PAO (0, 20, 50, 100 nM) for 24 hours. (**c**) Immunoblot analysis of Smad2/3, and GAPDH in lysates from BEAS-2B cells that were treated with NETs for 24 hours. (**d**) Densitometry Smad2/3 analysis from cell treated as depicted in (c). Data are presented as mean ± SEM (*n*=3), *P <0.05, ANOVA, as compared control (vehicle) to PAO-treated cells.

To assess whether the aforementioned delayed lung injury effects directly depend on PAO toxicity, we treated human primary airway epithelial cells (BEAS-2B cells) with different concentrations of PAO (0, 20, 50, or 100 nM) for 24 hours. The representative immunoblot demonstrated concentration dependent gradual increase in the expression levels of IL-33 (Fig. 5a–b), which were decreased at higher concentration of 100nM PAO. Furthermore, PAO treatment did not affect the expression levels of SAMD2/3 (Fig. 5c–d).

### PAO-induced NETs trigger upregulation of SMAD2/3 and IL-33 in primary human airway epithelial cells.

**Figure 6:**
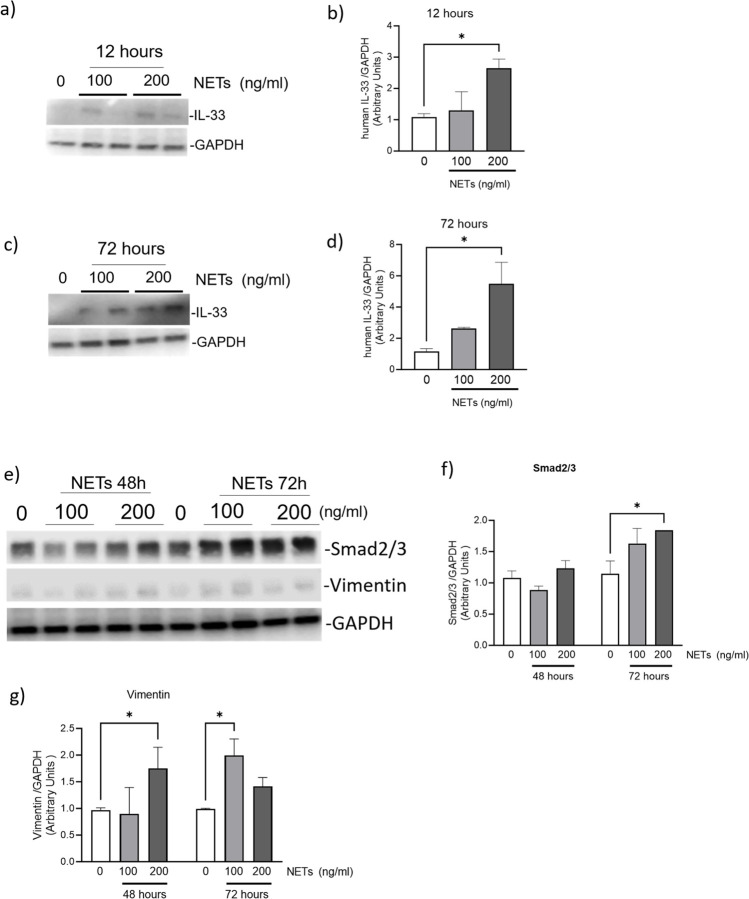
NETs-induced IL-33 secretion and EMT of BEAS-2B cells. (**a-d**) Immunoblot analysis of hIL-33 and GAPDH in lysates of BEAS-2B cells that were treated with NETs (0, 100 or 200 ng/ml) for 12 or 72 hours. The levels of hIL-33 is normalized to the loading control GAPDH (right panel). (**e-g**) Smad2/3, vimentin and GAPDH levels in cell lysates from untreated (control) BEAS-2B cells or after exposure to PAO for indicated time. All data are presented as mean ± SEM, (*n*=3). *P <0.05, ANOVA.

We performed dose-dependent and time-dependent studies on primary airway epithelial cells to determine the possible mechanism of induced inflammation and air-ways remodeling. The effects of NETs were assessed in the early (12 hours) and later (72 hours) time of injury, with low and high doses of NETs (100 and 200 ng/ml) on BEAS-2B cells. The treatment with NETs could induce the expression of IL-33 at 12 hours (Fig. 6a–b). However, the 72-hours treatment demonstrated a robust increase in the expression of IL-33 (Fig. 6c–d).

Furthermore, we determined the effects of chronic treatment with NETs on the expression of the EMT signaling regulator SMAD2/3 (Fig. 6e–f). The chronic treatment of NETs for 48 and 72 hours demonstrated increased expression levels of SMAD2/3 (Fig. 6e–f). The EMT marker and vimentin expression were also upregulated in NET-treated BEAS-2B cells (Fig. 6e and 6g).

## Discussion

The respiratory system is the most susceptible organ for arsenical-induced systemic injury. Though Lewisite was not used in the World Wars, its production, transportation, and military tests led to exposure in military personnel, and its delayed effects were manifested in years later [14]. Hence, the U.S. Department of Veterans Affairs offered healthcare aid to the veterans who were exposed to Lewisite during their service [14]. Sulfur mustard is an arsenic-based CWA which was used in Iran-Iraq War in 1980s. The follow-up clinical reports documented that 45% of the sulfur mustard survivors had delayed respiratory complications [15]. Hence, it is now established that the vesicant organoarsenicals cause delayed pulmonary injury in humans. Due to the paucity of studies in this field, we developed a novel murine model to further understand the pathological effects. Our study revealed that a single cutaneous exposure of PAO can cause ALI at 6 hours [16], and DLI at 10 weeks in mice [7]. In this chronological study, we aimed to understand single-cutaneous PAO-induced inflammation, its resolution, and the persistence of inflammation over the course of the acute and delayed effects in mice. We observed that all the PAO-exposed groups, i.e., 6, 24 hours, 1 and 10-week groups, demonstrated increased neutrophils and neutrophil elastase in the lung tissue.

The role of neutrophils in the pathogenesis of ARDS/ALI is indisputable [17]. We and others have demonstrated the crucial role of NETs in different animal models of ALI [2, 4, 5]. It is recognized that the neutrophil numbers play a crucial role in predicting the survival of ARDS patients, as a decreased count at the 24-hours mark signifies survival. In contrast, a persistent high count indicates a grim prognosis [3]. The neutrophil-to-lymphocyte ratio is a predictor for the survival of ARDS patients. A clinical retrospective study showed that neutrophils-to-lymphocyte ratio (NLR) could predict fatal complications in ARDS patients with COVID-19. The lower NLR value was associated with a survival advantage in the ARDS patients with COVID-19 [18]. Our data demonstrate that even a non-lethal dose of PAO, when administered on the skin, led to acute lung injury within 6 hours with the increased presence of neutrophils. Interestingly, 24 hours later, there was a notable decrease in neutrophils and neutrophil elastase. These findings indicate that the reduced number of neutrophils at 24 hours post exposure aided in the survival of the mice following PAO-induced acute lung injury.

NETs are generated by oxidative stress during inflammation. Our previous study demonstrated that PAO-induced netosis depended on the immediate Ca^+ 2^ influx pathway [16], rather than the NADPH-oxidase-dependent pathway [19]. PAO is an organic arsenic compound which is cleared from the system in a few days (ATSDR)[20]. It is understood that PAO exposure-mediated netosis is short-lived and should have led to a depletion of neutrophils and NETs in the lungs after a week. However, we observed an increased number of neutrophils and NETs in the lungs of the PAO-exposed 10-week group compared to controls.

Hence, we investigated the levels of IL-33, a known mediator of NETs-airway crosstalk [21]. In this chronological study, we observed the increased levels of IL-33 in the lungs throughout the 10-week post-PAO exposure. IL-33 activates NADPH oxidase to produce ROS, and this ROS production in IL-33-primed neutrophils leads to netosis [22–24]. These data suggest that the IL-33-mediated oxidative stress can maintain the sustained presence of NETs in cutaneous PAO-exposed mice.

IL-33 is an ‘alarmin’ released due to tissue damage and lytic cell death [13]. The chromatin fibers and neutrophil elastase increase the activation and secretion of IL-33 [6]. The secretion of IL-33 in acute respiratory distress syndrome (ARDS) is associated with worse outcomes [25]. At the same time, chronic release is linked to diseases of autoimmunity [26] and airway hyperresponsiveness [22, 27]. Given the close association between NETs and IL-33, we analyzed the expression levels of IL-33 in the lungs of mice exposed to PAO cutaneously and observed a significant increase in IL-33 levels in all the PAO-exposed groups as compared to controls, particularly highest at the 6-hour time point. PAO is a lipophilic and alkylating agent, which is very cytotoxic to the cells. PAO-mediated acute injury and cell death can cause an increase in IL-33 levels. IL-33 can perpetuate inflammation by increasing the infiltration of neutrophils and NETosis [22, 26, 28]. NETs exacerbated the inflammation through IL-33 [28]. At 24 hours, a decrease in PAO-mediated neutrophils, neutrophil elastase, and IL-33, was observed compared to the 6-hour time point, serving as biomarkers for the survival of mice from PAO-induced acute lung injury. However, the decreased levels of IL-33 and NETs remained significantly higher in PAO-exposed mice when compared to control mice, indicating the persistent presence of NETs leading to upregulated levels of IL-33 in the lungs, or *vice-versa*. It is previously known that IL-33 secretion from airway epithelial cells increases neutrophil recruitment and NETosis in airways [7, 8]. NETs are an important factor in enhancing the bioactivity of IL-33 [29]. The proteases on NETs can cleave the full-length IL-33 into its more potent and bioactive form [29]. NETs and IL-33 complexes exert type I IFN-mediated autoimmune inflammation [26]. Additionally, TLR2, which DAMPs can activate, can induce the IL-mRNA of IL-33 in a dependent TRAF6 and IRF7-dependent manner [30]. IRF7 can be activated by self-DNA [31]. Based on these studies and our data, we postulate that NETs increase the IL-33 expression through IRF7 signaling. In addition, IL-33 induces the production of Th2 cytokines, promoting the development of Th2-related airway remodeling and inflammation [32]. As a future direction for this study, we will investigate the exact mechanisms of IL-33 and NETs interactions for the feedforward cycle of systemic and persistent inflammation in cutaneous arsenical induced airway disease.

Airway remodeling includes increased airway epithelial cell injury, inflammation, increased ECM production, and pro-fibrotic EMT signaling. NETs promote pro-fibrotic signaling, and the transgenic mice model of NETs deficiency are protected from bleomycin-induced lung fibrosis [33]. Additionally, studies have demonstrated that in vitro NETs treatments trigger EMT, and TGFβ1 secretion [34]. Our results also demonstrated increased SMAD2/3 and increase in the expression of EMT markers vimentin in the BEAS-2B cells. IL-33 expression are elevated by cell damage, and chromatin binding, hence, as expected, PAO and NETs both were capable of increasing the IL-33 expression levels. Interestingly, direct PAO treatment did not increase the SMAD2/3 expression. It may be due to it’s ability to inhibit endocytosis. PAO reacts with vicinal sulfhydryl groups, forming stable ring structures, and is the inhibitor of receptor-mediated endocytosis. A report demonstrated that treatment with PAO inhibited the endocytosis of TGFβ1, which inhibited the activation of SMAD2/3 [9]. It is demonstrated that endocytosis of TGFβ1 and TGFβ1 receptors is essential for SMAD-mediated EMT signaling.

## Conclusions

A sub-lethal dose of PAO triggers acute lung injury in the early phase through neutrophilic and NETs-mediated mechanisms. During the resolution of inflammation, levels of neutrophils and NETs are reduced but not completely eliminated. The continuous presence of the NETs-IL-33 axis may contribute to airway remodeling 10 weeks after PAO exposure. While PAO itself is unable to initiate pro-fibrotic EMT signaling in human primary airway epithelial cells, it does increase IL-33 levels. Overall, PAO-induced NETs have a direct effect on airway remodeling by increasing IL-33 and SMAD 2/3 signaling. Our data suggest that PAO and NETs-mediated increased IL-33 signaling perpetuate a feed-forward cycle of NETs generation. However, only NETs are responsible for the pro-fibrotic signaling in bronchial airway epithelial cells. The dysregulated NETs-IL-33 responses disrupt the immune response and sustain persistent neutrophil recruitment to the lungs, perpetuating the cycle of inflammation. Our model indicates that local and systemic damage to lung tissue by PAO and subsequently generated NETs are responsible for delayed lung injury. Our study suggests that NETs-targeting approaches mitigate airway remodeling in organoarsenical induced delayed lung injuries.

## Methods

### Animal Exposure to phenyl arsine oxide.

All animal protocols were approved by the Institutional Animal Care and Use Committee at the University of Alabama at Birmingham (UAB). PAO (MilliporeSigma, catalog P3057) exposures were performed on C57BL/6 at the University of Alabama animal facility. Mice were purchased from The Jackson Laboratory. PAO was applied once topically on the dorsal skin of mice on a 2.56 cm^2^ skin area, as described [7]. Briefly, mice were anesthetized with 100 mg/kg of ketamine and 5–7 mg/kg of xylazine injection i.p., and 0.05–0.1 mg/kg buprenorphine as an analgesic. Shaved 8- to 12-week-old male and female C57BL/6 mice were treated with PAO topically (150 μg/mouse diluted in 30 μL ethanol and applied over 2.56 cm^2^ skin area). Mice were sacrificed at 6 hours, 24 hours, 1 week, and 10-week time points. The lungs were harvested for further assessment as previously described [7].

### ELISA for Cit-H3 and IL-33 measurements.

Lungs homogenates obtained from animals of each group and assessed Cit-H3 (Cell Signal Technology,), and mouse IL-33 (Abcam) measurements in the lung tissue according to the manufacturers’ specifications.

### Immunoblot analysis.

Whole-lung lysates were processed for immunoblotting according to standard procedures [16] using the following primary antibodies: neutrophil elastase (Cell Signaling Technology, Cat. # 90120), anti-GAPDH-HRP antibody (Proteintech, Cat. # HRP-60004), SMAD2/3 (Cell Signaling Technology, Cat. # 8685), and IL-33 (Cell Signaling Technology, Cat. # 88513S). Briefly, after electrophoresis on 4–20% gels (Bio-Rad), proteins were transferred to a PVDF membrane (Bio-Rad). Membranes were blocked, incubated with primary antibodies, and exposed to HRP-conjugated anti-rabbit or anti-mouse antibodies (MilliporeSigma, catalog AP187P, and AP130). KwickQuant-Pro imager system was used for the image acquisition, and band intensities were quantitated using ImageJ (NIH).

### Histopathological examination and immunohistochemistry. .

The H&E staining procedure was performed following the method described [16]. In summary, the lungs were fixed in 10% buffered formalin and then embedded in paraffin. Tissue sections of 5 μm thickness were obtained using a microtome (HM 325, Thermo Fisher Scientific). These sections were deparaffinized in xylene and then rehydrated. At least 3 independent tissue sections from each group underwent H&E staining and were examined for histological changes using a Keyence microscope.

Immunohistochemistry of the lungs for anti-NE, anti-Cit-H3, anti-α-SMA, and anti-Collagen I was carried out as previously described. The lung section was immunostained for anti-NE (Proteintech, dilution 1:500), anti-Cit-H3 (Abcam, dilution 1:300), anti-α-SMA (Santa Cruz Technology, 1:250), and anti-Collagen I (Rockland Immunochemicals, dilution 1:500) overnight.

### Picro-Sirius Staining.

Paraffin-embedded lung sections were deparaffinized [7] and stained for Picro-Sirius using a Picro Sirius staining kit (Abcam) as per the manufacturer’s instructions.

### Treatments of Human Primary Airway Epithelial with NETs.

The concentrated (5 times) NETs (100 ng/mL) from the supernatant of neutrophils were isolated as described [16] and incubated with BEAS-2B cells (Lonza). A total of 5 × 10^5^ cells were seeded per well in the 6-well plate for various time points according to the experiments. Cell were maintained in the specialized media The cell lysate was prepared using RIPA buffer supplemented with a Protease Inhibitor cocktail (ThermoFisher) as described [16].

### Statistical Analysis.

Statistical analyses were performed using GraphPad Prism Software (Version 8.0). Data are presented as means ± SEM. Significance was determined using the unpaired Student’s t-test (n ≥ 3 for cell culture studies; n = 3–6 mice/group). Statistical significance was as follows: (ns) non-significant, *p < 0.05, **p < 0.01, and ***p < 0.001 compared to control group scores.

## Supplementary Material

Supplement 1

## Data Availability

Data is provided within the manuscript or supplementary information files.
