# Supplementary material for: Persistent Neutrophilic Inflammation is Associated with Delayed Toxicity of Phenylarsine Oxide in Lungs
Source: Res Sq. 2025 Jan 13:rs.3.rs-5100050. Preprint. [Version 1] doi: 10.21203/rs.3.rs-5100050/v1 (PMC11774458; doi:10.21203/rs.3.rs-5100050/v1)
Supplement: Supplement 1 [file NIHPPRS5100050v1-supplement-1.pdf]

Supplementary Files

This is a list of supplementary files associated with this preprint. Click to download.

- WBoriginalsandmembranesuploadedversion10124.pdf
